# Disparities in the association between ambient temperature and preterm birth according to individual and regional characteristics: a nationwide time-stratified case-crossover study

**DOI:** 10.1186/s12940-024-01062-6

**Published:** 2024-02-22

**Authors:** Jieun Min, Whanhee Lee, Jongmin Oh, Youngrin Kwag, Eunji Kim, Joyce Mary Kim, Kyung A Lee, Eunhee Ha

**Affiliations:** 1 Department of Environmental Medicine, College of Medicine, Ewha Womans University, Seoul, Republic of Korea; 2 Graduate Program in System Health Science and Engineering, College of Medicine, Ewha Womans University, Seoul, Republic of Korea; 3 School of Biomedical Convergence Engineering, College of Information and Biomedical Engineering, Pusan National University, Yangsan, Republic of Korea; 4 Institute of Ewha-SCL for Environmental Health (IESEH), SCL for Environmental Health (IESEH), Ewha Womans University College of MedicineEwha Womans University College of Medicine, Seoul, Republic of Korea; 5 Department of Human Systems Medicine, College of Medicine, Seoul National University, Seoul, Republic of Korea; 6 Department of Obstetrics and Gynecology, College of Medicine, Ewha Womans University, Seoul, Republic of Korea; 7 Department of Medical Science, Ewha Womans University School of Medicine and Ewha Medical Research Institute, Seoul, Republic of Korea

**Keywords:** Ambient temperature, Preterm birth, Disparity, Urbanicity, Greenness, Medical resources

## Abstract

**Background:**

Several studies have reported that climate change elevates heat exposure in pregnant women and high temperatures during pregnancy are associated with preterm births (PTBs). Although the association might be disproportionate, related evidence remains sparse. We evaluated the disproportionate risk of PTB associated with ambient temperature during pregnancy by individual and regional characteristics in South Korea.

**Methods:**

We collected data on birth certificates and daily mean temperatures during the period from 2011 to 2019. A time-stratified case-crossover design was used to investigate the association between temperature and PTB and stratified analyses were conducted to examine the effect modification of individual and regional characteristics.

**Results:**

A total of 160,067 singleton PTBs were recorded in Korea from 2011 to 2019. A 5℃ increase in the mean temperature during the last four weeks before delivery was associated with an increased risk of PTB with an odds ratio (OR) of 1.03 (95% confidence interval [CI]: 1.02, 1.05), and the association was more evident in mothers aged ≥35 years (OR: 1.06 [95% CI: 1.03, 1.10]) and with low education levels (OR: 1.04 [95% CI: 1.02, 1.05]). Additionally, the estimated risk was evident in districts with lower medical resources and more prominent disparities were shown by individual and regional characteristics in rural areas than in urban areas.

**Conclusions:**

This study provides evidence that the risk of PTB related to ambient temperature is disproportionate by individual and regional characteristics and suggests the need for public health policies to alleviate the disparities, especially in rural areas.

**Supplementary Information:**

The online version contains supplementary material available at 10.1186/s12940-024-01062-6.

## Introduction

Global warming has been considered a threat to human health as well as an environmental emergency leading to concern for the necessities of life [1, 2]. Increasing temperature is associated with women`s health and pregnant women are an especially at-risk population vulnerable to the adverse health effects [3, 4]. Emerging evidence suggests that high ambient temperatures are also associated with adverse pregnancy outcomes such as preterm birth (PTB) and low birthweights [5–7].

Among the adverse birth outcomes, PTB, defined as the birth of an infant before less than 37 completed weeks, is a leading global cause of neonatal mortality and morbidity in children under five years of age [8]. The World Health Organization has estimated that in 2019 nearly 1 in 10 births were preterm, which equated to almost 13.4 million births worldwide [9]. Sequelae of PTB, such as respiratory distress syndrome and bronchopulmonary dysplasia, can create cascading and interrelated neurodevelopmental disability, social and emotional problems, learning difficulties, and cognitive impairment throughout childhood continuing even into adulthood, thereby leading to high incurred cost burdens for care [8].

Since it is important to identify the determinants of PTB for prevention and control, previous studies have tried to establish a causal association between potential risk factors and PTB. Although previous studies have investigated the associations between PTB and high ambient temperature, discrepancies remain across these studies [4, 10–12]. These varying results could be related to numerous contributing factors (at the individual or regional level), which modify the effect of ambient temperature on PTB, including maternal age, maternal socioeconomic status, regional urbanicity, and greenness. Only a few studies have investigated the role of greenness and medical resources as an effect modifier on associations between heat exposure during pregnancy and PTB, but their findings were inconclusive [13–16]. Furthermore, women living in rural areas are more likely to face health disparities compared to those in urban areas because of the geographical isolation, limited healthcare resources, and limited job opportunities in rural areas [17]; thus, the disproportionate risk of PTB associated with the ambient temperature needs to be investigated within both rural and urban areas. To the best of our knowledge, the effect modification of medical resources in the association between temperature and PTB has been rarely investigated in previous studies and there is no research on the individual and regional disparities concerning temperature-related PTB risk within each rural and urban area. Thus, identifying potential effect modifiers in the association between ambient temperature and PTB and examining the related disparities within rural and urban areas can provide meaningful evidence for public health to manage PTB-related inequality.

Therefore, the aim of this study is to estimate the association between ambient temperature during pregnancy and PTB by applying a time-stratified case-crossover design using a nationwide registry and to identify individual and regional characteristics leading to disparities in the risk of PTB associated with the ambient temperature within the rural and urban areas of South Korea (hereafter, Korea).

## Methods

### Study design and population

This study applied a time-stratified case-crossover design to investigate the association between exposure to ambient temperature during pregnancy and PTB in Korea. This design is a case-only within-person comparison; therefore, there is no need to adjust for unmeasured time-invariant individual characteristics [18]. Each PTB case was matched to three or four controls on the same day of the week in the same month (Fig. S1).

**Fig. 1:**
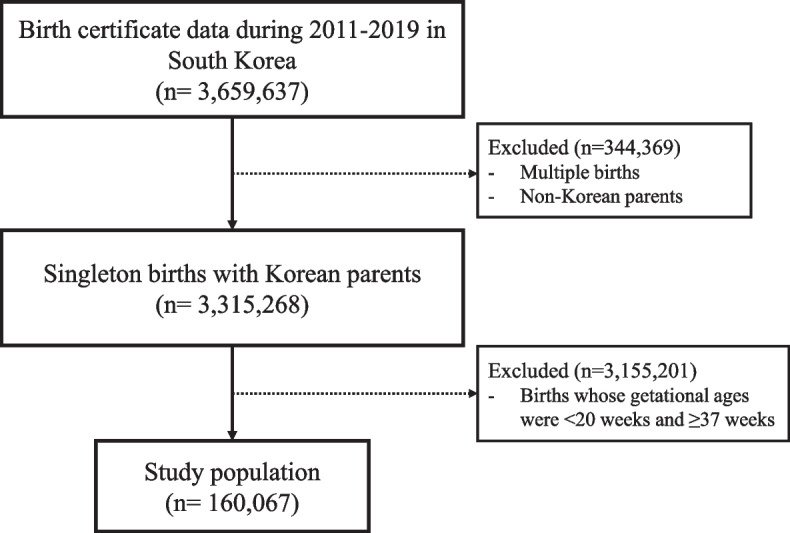
Flow chart of this study

We collected birth certificate data from the Korea National Statistical Office from January 1, 2011 to December 12, 2019. The data included the date of each birth, the mother’s residential address, the infant’s sex, gestational age, parity, maternal age, and education level. Of the total 3,659,637 births in Korea during the study period, we restricted the study participants to 3,315,268 singleton births where the nationality of the parents was Korean. PTB was defined as a live birth whose gestational age was between ≥ 20 weeks and < 37 weeks. Overall, 160,067 PTBs were included in the analysis (Fig. 1). In addition, we defined the following sub-categories of PTB based on gestational age: late (34 to 37 weeks), moderate (32 to 34 weeks), very (28 to 32 weeks), and extreme (20 to 28 weeks) PTB [19].

### Exposure variables

We obtained data on the daily mean temperature for all 229 districts (corresponding ZIP code spatial resolution of the USA) of Korea for the period of 2011–2019 from the Korean Meteorological Office. Real-time hourly temperature data were provided at the level of “dong,” which is a smaller administrative region than a district. The daily mean temperature was averaged across all dongs for each day in each of our study districts. We also collected the particulate matter ≤10μm (PM_10_) concentration from the Korea Ministry of Environment to adjust for in the model, because an increased risk of PTB was associated with exposure to PM_10_ [20]. The PM_10_ concentration was measured hourly from single or multiple monitoring stations for each district. For districts with multiple monitors, we averaged monitor-specific values to generate district-level concentrations. Daily PM_10_ concentrations were averaged across the monitoring station for each day.

### District-level indicators

We collected six district-level indicators during the study period. These variables partly explain the (1) urbanization (population density), (2) greenness (park area per person and enhanced vegetation index [EVI]), and (3) medical resource (# obstetrics and gynecology [OB/GYN] specialists per 1,000 persons, emergency room [ER] utilization rate within standard time, and delivery room [DR] utilization rate within standard time) of each district. Using a population density variable, we defined rural and urban areas based on a median value of the population density according to previous studies [21]. We chose EVI as a greenness indicator instead of a normalized difference vegetation index (NDVI) in this study because the EVI is similar to the NDVI, but it additionally corrects for some atmospheric conditions and canopy background noise [22]. We could not obtain an EVI of three island districts because there were limitations in collecting reliable remote sensing data. The park area per person was included in the study as a means of measuring the actual available greenspace. The ER and DR utilization rates within standard time (%) were specified as the percentage of utilization within standard time (60 minutes) out of the total ER and DR utilization by the local residents, respectively. The lower the utilization rate within the standard time, the more vulnerable it was. Detailed descriptions of the definition and data sources are presented in Table S1 . All district-level indicators were averaged across the study period.

### Statistical analysis

The case-crossover design was conducted using a conditional logistic regression model within the same subjects. To estimate the association between temperature and PTB, the moving average of the daily mean temperature during the last four weeks before delivery was linearly included in the model individually and the results were presented as the odds ratio (OR) for a 5℃ increase in the moving average of temperature. In addition, the current day’s PM_10_ concentration was linearly included in the model.

To examine the disproportionate association between ambient temperature and PTB by individual and regional characteristics, we performed sub-population and sub-district analyses by repeating the main model for each subgroup. The study population was divided into sub-populations using maternal age (<35 years or ≥35 years), parity (primiparity or multiparity), maternal education level (≤ college or > college), and classification of prematurity (late to moderate preterm or very to extreme preterm). We also categorized regional indicators: the population density was used to specify urbanization (rural or urban) based on the median value and the rest of the five district-level indicators which partly explain greenness and medical resources were divided into three categories (low, mid, and high) based on the 33.3 and 66.6 percentile for each variable. In addition, we conducted double stratification as follows: urbanization (rural and urban) $\times$ individual characteristics (maternal age, parity, maternal education level, and classification of prematurity) and urbanization $\times$ regional characteristics (greenness and medical resources). For the double stratification, greenness and medical resources were categorized into two sub-districts (low and high) as above or below the median.

To evaluate the robustness of our results, we also conducted sensitivity analyses. First, we applied different exposure periods to the ambient temperature (three or five weeks before delivery). Second, we performed the model without adjusting for PM_10_ concentrations. All statistical analyses were conducted using the R statistical software, version 4.1.0.

## Results

A total of 160,067 singleton PTBs were recorded and the proportion of PTBs among the total number of singleton births was 4.8% during the period of 2011–2019 in Korea (Table 1). The proportion was higher in mothers aged ≥35 years, multiparity, and low education levels compared to mothers aged <35 years, primiparity, and high education levels, respectively. The time trend of the proportion of PTBs (Fig. S2) showed increasing incidence over the study period and a seasonal pattern of PTB. The mean temperature was 12.81℃ in Korea during the study period and it ranged from 8.84℃ to 16.45℃ among 229 districts (Table S2). The spatial distribution of the district-level mean temperature and PM_10_ concentration during the study period is presented in Fig. S2. More than half of the PTBs occurred in urban areas (*n* = 132,378) as compared to the rural areas (*n* = 27,689) (Table S3). The number of PTBs according to individual and regional characteristics in the rural and urban areas are presented in Table S4.

**Table 1:** Baseline characteristics of the study population (preterm births, PTBs) during 2011–2019 in South Korea

	PTB (%)^a^	Proportion of PTB (%)^b^
**Overall**	160,067 (100)	4.83
**Year**		
2011	19,046 (11.90)	4.42
2012	20,192 (12.61)	4.57
2013	18,426 (11.51)	4.64
2014	18,762 (11.72)	4.75
2015	19,311 (12.06)	4.84
2016	18,109 (11.31)	4.92
2017	16,819 (10.51)	5.22
2018	15,171 (9.48)	5.19
2019	14,231 (8.89)	5.30
**Maternal age**		
<35 years	112,324 (70.2)	4.46
≥35 years	47,743 (29.8)	6.00
**Parity**		
Primiparity	80,319 (50.2)	4.61
Multiparity	79,748 (49.8)	5.07
**Maternal education level**		
≤ College	148,106 (92.53)	4.85
> College	10,970 (6.85)	4.48
Unknown	991 (0.62)	5.26
**Classification of prematurity**^**c**^		
Late	127,962 (79.9)	-
Moderate	14,708 (9.2)	-
Very	12,362 (7.7)	-
Extreme	5,035 (3.1)	-

^a^Column percent

^b^Proportion of PTB was calculated by dividing the number of PTB by total number of singleton births.

^c^Classification of prematurity was specified based on gestational age: late (34 to 37 weeks), moderate (32 to 34 weeks), very (28 to 32 weeks), and extremely (20 to 28 weeks) preterm birth.

Table 2 shows the association between the ambient temperature and PTBs by individual characteristics. The OR for PTB associated with a 5℃ increase in the moving average of the temperature during the last four weeks before delivery was 1.03 (95% confidence interval [CI]: 1.02, 1.05). The association was more evident in mothers aged ≥35 years (OR: 1.06) and with a low education level (OR: 1.04) than in mothers aged <35 years (OR: 1.02) and with a high education level (OR: 1.00). As we altered the exposure time window of the ambient temperature to three or five weeks before delivery, the results were generally consistent (Table S5) and these findings were also robust when the PM_10_ was excluded from the model (Table S6).

**Table 2:** Association between ambient temperature during the last four weeks before delivery and preterm birth by individual characteristics

	OR (95% CI)		
	Overall	Rural	Urban
**Overall**	1.03 (1.02, 1.05)	1.06 (1.01, 1.12)	1.03 (1.01, 1.05)
**Maternal age**			
<35 years	1.02 (1.00, 1.04)	1.04 (0.98, 1.10)	1.02 (1.00, 1.04)
≥35 years	1.06 (1.03, 1.10)	1.12 (1.02, 1.23)	1.06 (1.02, 1.09)
**Parity**			
Primiparity	1.03 (1.01, 1.06)	1.11 (1.03, 1.19)	1.02 (1.00, 1.05)
Multiparity	1.03 (1.01, 1.06)	1.01 (0.93, 1.10)	1.03 (1.00, 1.06)
**Maternal education level**			
≤ College	1.04 (1.02, 1.05)	1.06 (1.01, 1.12)	1.03 (1.01, 1.05)
> College	1.00 (0.94, 1.07)	1.09 (0.87, 1.36)	0.99 (0.93, 1.06)
**Classification of prematurity**^**a**^			
Late to moderate preterm	1.03 (1.01, 1.05)	1.08 (1.02, 1.13)	1.03 (1.01, 1.05)
Very to extreme preterm	1.03 (0.98, 1.08)	0.97 (0.83, 1.12)	1.04 (0.99, 1.10)

ORs were presented as those for 5℃ increase in moving averaged-temperature during the last four weeks before delivery. Urban and rural were categorized using median of population density.

^a^Classification of prematuriy was specified based on gestational age: late to moderate preterm (32 to 37 weeks) and very to extreme preterm (20 to 32 weeks).

*CI* confidence interval, *OR* odds ratio

**Fig. 2:**
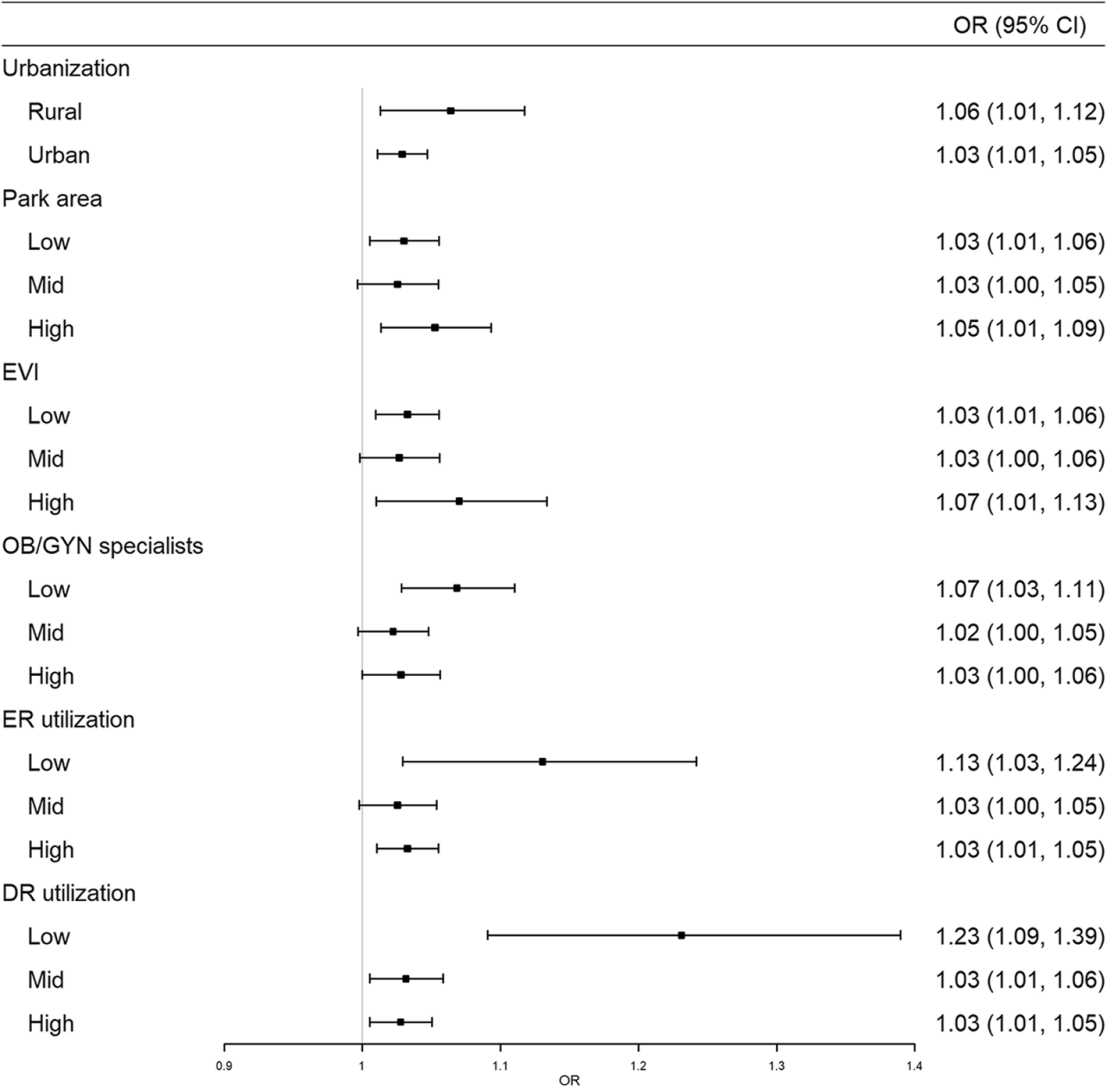
Association between ambient temperature during the last four weeks before delivery and preterm birth by regional characteristics. Odds ratios (ORs) were presented as those for 5℃ increase in moving averaged-temperature during the last four weeks before delivery. Urban and rural were categorized using median of population density. OB/GYN=obstetrics and gynecology, ER=emergency room, DR=delivery room

Figure 2 displays this association by regional characteristics. The association between the ambient temperature during pregnancy and a PTB was higher in rural areas compared to urban areas: OR for PTB associated with a 5℃ increase in the moving averaged-temperature during the last four weeks before delivery was 1.06 (95% CI: 1.01, 1.12) in rural areas and 1.03 (95% CI: 1.01, 1.05) in urban areas. In addition, the risk of PTB associated with temperature was prominent in districts with low medical resources (OB/GYN specialists per 1,000 persons and ER/DR utilization rate within standard time).

When we examined the disproportionate association between the ambient temperature and a PTB by individual characteristics within rural and urban areas, the risk of PTB in older mothers (≥35 years) and primiparity in rural areas was much higher than that of other populations (Table 2). In addition, the risk of PTB was not prominently different by prematurity classification in urban areas, while the risk of late to moderate PTB (OR: 1.08) was more evident than that of very to extreme PTB (OR: 0.97) in rural areas. We also investigated the association according to regional greenness and medical resources within rural and urban areas (Table 3). We found a higher risk of PTB in districts with higher greenness and lower medical resources in rural areas, while the risk showed no difference by greenness and medical resources in urban areas.

**Table 3:** Association between ambient temperature during the last four weeks before delivery and preterm birth by greenness and medical resources in rural and urban area

	OR (95% CI)	
	Rural	Urban
**Enhanced vegetation index**		
Low	1.07 (1.00, 1.14)	1.03 (1.01, 1.06)
High	1.09 (1.01, 1.17)	1.02 (1.00, 1.05)
**Park area per person**		
Low	1.04 (0.97, 1.11)	1.03 (1.01, 1.06)
High	1.09 (1.02, 1.16)	1.03 (1.00, 1.05)
**OB/GYN specialists per 1,000 persons**		
Low	1.09 (1.00, 1.18)	1.03 (1.00, 1.05)
High	1.05 (0.99, 1.16)	1.03 (1.01, 1.06)
**ER utilization rate within standard time**		
Low	1.07 (0.98, 1.15)	1.03 (1.00, 1.06)
High	1.06 (1.00, 1.13)	1.03 (1.00, 1.05)
**DR utilization rate within standard time**		
Low	1.12 (1.04, 1.22)	1.03 (1.01, 1.06)
High	1.03 (0.98, 1.10)	1.02 (1.00, 1.05)

ORs were presented as those for 5℃ increase in moving averaged-temperature during the last four weeks before delivery. Urban and rural were categorized using median of population density. Greenness and medical resource indicators were categorized using median of each variable.

*CI* confidence interval, *DR* delivery room, *ER* emergency room*, OB/GYN* obstetrics and gynecology, *OR* odds ratio

## Discussion

With a nationwide registry including 160,067 PTBs out of 3,315,268 singleton births in Korea between 2011 and 2019, we identified a positive association between maternal exposure to ambient temperature and PTB, which was more evident in mothers aged ≥35 years and with a low educational level. Furthermore, the disproportionate association was observed by regional characteristics; a higher risk of PTB due to an increase in ambient temperature in rural areas and districts with low medical resources was seen. Compared to urban areas, the risk was more disproportionate by individual and regional characteristics in rural areas.

We found that an increase in ambient temperature was associated with PTB, which was consistent with evidence from the previous study [23]. Previous literature has reported the impacts of heat or heat wave exposure during pregnancy on PTB. One study from a city in China suggested that a sharp increase in temperature could be a risk factor for PTB independent of the mean daily temperature [23]. The association between exposure to high temperature during pregnancy and the risk of PTB can be explained through several biological mechanisms. High temperature-related dehydration increases blood viscosity, elevates cholesterol levels and reduces uterine blood flow in pregnant women, which can induce uterine contractions and the onset of labor [24, 25]. In addition, animal studies have shown that heat exposure may increase the secretion of proinflammatory cytokines, such as prostaglandin F2a (PGF2a), and oxytocin to induce labor [26].

In our study, we focused on the temperature rise during four weeks before delivery. Previous studies have suggested the critical exposure window for high temperature exposure and PTB, but have not identified consistent exposure time frames [27–30]. A recent study conducted in a city in China on the evaluation of time windows reported associations between extremely and moderately high temperatures and PTB during the 4-week time window but not the 1-week time window [28].

We observed that the association between ambient temperature and PTB was more evident in pregnant women ≥35 years of age and with a low education level. An increased risk of PTB among older women (≥35 years) is induced by medically indicated PTB, considering that advanced maternal age is a contributing factor for medical conditions, such as preeclampsia and fetal growth restriction, whereby the impact of exposure to high temperature is potentially suggested in the literature [6, 31]. On the other hand, given that advanced maternal age (≥35 years) was suggested as an independent risk factor for spontaneous preterm labor, mechanisms for spontaneous PTB, such as infection, stress, uterine overdistension, decidual senescence, cervical disease, vascular disorders, and breakdown of maternal-fetal tolerance, should not be excluded [32, 33]. Moreover, our findings showed that the maternal education level can modify the association between temperature and PTB. Previous studies have provided consistent evidence of an increased risk of PTB from heat in mothers with low socioeconomic statuses (e.g., household income and education) [13, 34]. Consequently, we postulated that pregnant women with low socioeconomic status might be more susceptible to heat due to a lack of adequate nutrition or low access to medical care.

In addition, individual and regional disparities related to the association between ambient temperature and PTB were more prominent in rural than urban areas. In rural areas, the risk was higher in the sub-populations of primiparity and late to moderate PTB compared to multiparity and very to extreme PTB, while the risk differences by these individual characteristics were not considerable in urban areas. There might be different underlying mechanisms in early, very, and extreme preterm, like intraamniotic infection or inflammation, vascular placental diseases and others [35]. Recent evidence has indicated that the rates of morbidity and mortality are greater in late preterm newborns compared to those in term ones [36, 37]. Therefore, we hypothesized that an appropriate treatment for late to moderate PTB is important; however, as resources for this are insufficient in rural areas, the risk of late to moderate PTB due to ambient temperature is higher in rural areas. On the other hand, several very to extreme PTBs may have led to stillbirths; thus, we could not identify the case from the birth certificate data, which resulted in an underestimation of the risk of very to extreme PTBs attributable to temperature in this study. Taken together, a healthcare focus on prematurity should also consider late-to-moderate preterm births.

We also determined that the risk was higher in districts with high greenness and low medical resources in rural areas, while the risk difference by these residential characteristics was not considerable in urban areas. Currently, increasing evidence indicates that vegetation influences ambient temperature through transpiration, shading, and modified convection; green spaces may buffer heat islands within urban areas [13, 24, 38–41]. Apart from the role that greenness has in reducing ambient temperature, other possible pathways that may reduce the risk of PTB include the salutogenic effects provided by reducing stress, psychological restoration, and opportunities for increased physical activity. However, in rural areas, where island effects were unlikely to occur, we observed higher associations between temperature rise PTB in districts with high greenness (high park area per person and high EVI). For pregnant women living in rural areas, green spaces might be workplaces rather than places for rest and relaxation. Moreover, the association between ambient temperature during pregnancy and PTB was higher in medically vulnerable districts (i.e., districts with low OB/GYN specialists per 1,000 persons and ER and DR utilization rates within standard time) in rural areas, while the risk did not differ by medical resources in urban areas. This finding can justify the need for monitoring pregnant women in rural areas located far from health facilities that can provide medical services for the prevention and treatment of preterm labor and thus PTB.

To the best of our knowledge, this study is the first study to examine the individual and regional disparities in the association between ambient temperature during pregnancy and PTB. The main strength of our study was the use of high-quality nationwide data on exposure and outcome, birth certificate data from the Korea National Statistical Office, data from the Korean Meteorological Office, and a database of community health outcomes and determinants distributed by the Korea Disease Control and Prevention Agency (KDCA). In this large-scale and population-based cohort study, we showed the main effect of ambient temperature increase during the last four weeks before delivery on PTB and comprehensively investigated the individual and regional disparities within rural and urban areas.

A few limitations should be considered when interpreting the findings of our study. First, exposures were assigned based on the maternal residential address reported on the infant birth certificate, which could increase exposure misclassification as we did not account for residential mobility and time-activity patterns of the mother. Future studies may include the maternal addresses prior to and throughout the pregnancy and use the global positioning system and personal monitors to examine real-time personal-level green space and air pollution exposures. Another limitation of using the administrative dataset was the lack of information on detailed clinical characteristics, individual lifestyle and behavioral factors. To overcome this problem, we selected a case-crossover design, which has been extensively applied in the literature to investigate the associations between short-term exposures to environmental determinants and health outcomes, including temperature and PTB. The key strength of the design was that each case served as its own control, which implies a perfect adjustment for the observed or unobserved individual-level confounders that do not vary, or vary slowly, over time, such as age, smoking habits, and socioeconomic deprivation. Finally, although the particulate matter ≤2.5μm (PM_2.5_) was known to be associated with PTB, we could not adjust for PM_2.5_ in the model because monitoring stations that measure PM_2.5_ were not established until 2015 and the number of stations was not large enough to cover all 229 districts in Korea. Thus, instead of using PM_2.5_, we adjusted for the daily mean concentration of PM_10_ to consider the potential confounding effect of air pollution.

## Conclusion

We found that maternal exposure to increased ambient temperature during the four weeks before delivery may increase the risks of PTB. Additionally, our findings demonstrate that the risk of PTB related to increased temperature can be disproportionate by the maternal individual (age and education level) and regional (urbanicity, greenness, and medical resources) factors, and related disparities were evident in rural areas. This study provides evidence that climate change can affect PTB by exposing pregnant women to increasing heat exposure and suggests a need for public health policies to alleviate related disparities, especially in rural areas.

### Supplementary Information

**Additional file 1: Table S1.** Detailed description on district-level indicators. **Table S2.** Descriptive statistics of exposures and regional indicators in 229 districts. **Table S3. **Number of preterm births by regional characteristics. **Table S4. **Number of preterm births by individual and regional characteristics in rural and urban. **Table S5.** Sensitivity analysis with different time window of exposure to ambient temperature. **Table S6. **Sensitivity analysis without PM_10_ adjustment in a conditional logistic model. **Fig. S1.** Time-stratified case-crossover design. **Fig. S2. **Time trend of proportion of preterm birth (PTB) during 2011–2019. **Fig. S3.** Spatial distribution of district-level mean temperature and PM_10_ concentration during 2011–2019.

## Data Availability

The dataset analyzed during the current study are available in the Korea National Statistical Office repository (https://kosis.kr/index/index.do), Korean Meteorological Office (https://data.kma.go.kr/cmmn/main.do), and the Korea Diseases Control and Prevention Agency (KCDA) (https://chs.kdca.go.kr/chs/main.do).

## Abbreviations

PTB: preterm birth
PM_10_: particulate matter ≤10μm
EVI: enhanced vegetation index
OB/GYN: obstetrics and gynecology
ER: emergency room
DR: delivery room
NDVI: normalized difference vegetation index
OR: odds ratio
CI: confidence interval
PM_2.5_: particulate matter ≤2.5μm
