# Supplementary material for: Disparities in the association between ambient temperature and preterm birth according to individual and regional characteristics: a nationwide time-stratified case-crossover study
Source: Environ Health. 2024 Feb 22;23:23. doi: 10.1186/s12940-024-01062-6 (PMC10882820; doi:10.1186/s12940-024-01062-6)
Supplement: Supplementary file 1 — Additional file 1: Table S1. Detailed description on district-level indicators. Table S2. Descriptive statistics of exposures and regional indicators in 229 districts. Table S3. Number of preterm births by regional characteristics. Table S4. Number of preterm births by individual and regional characteristics in rural and urban. Table S5. Sensitivity analysis with different time window of exposure to ambient temperature. Table S6. Sensitivity analysis without PM10 adjustment in a conditional logistic model. Fig. S1. Time-stratified case-crossover design. Fig. S2. Time trend of proportion of preterm birth (PTB) during 2011–2019. Fig. S3. Spatial distribution of district-level mean temperature and PM10 concentration during 2011–2019. [file 12940_2024_1062_MOESM1_ESM.docx]

**Supplementary materials**

**Title.** **Disparities in the association between ambient temperature and preterm birth according to individual and regional characteristics: a nationwide time-stratified case-crossover study**

**Authors.** Jieun Min, Whanhee Lee, Jongmin Oh, Youngrin Kwag, Eunji Kim, Joyce Mary Kim, Kyung A Lee^*^, Eunhee Ha^*^

**Affiliation**

^1^Department of Environmental Medicine, College of Medicine, Ewha Womans University, Seoul, Republic of Korea
^2^Graduate Program in System Health Science and Engineering, College of Medicine, Ewha Womans University, Seoul, Republic of Korea
^3^School of Biomedical Convergence Engineering, College of Information and Biomedical Engineering, Pusan National University, Yangsan, Republic of Korea
^4^Institute of Ewha-SCL for Environmental Health (IESEH), Ewha Womans University College of Medicine, Seoul, Republic of Korea
^5^Department of Human Systems Medicine, College of Medicine, Seoul National University, Seoul, Republic of Korea
^6^Department of Obstetrics and Gynecology, Ewha Womans University College of Medicine, Seoul, Republic of Korea
^7^Department of Medical Science, Ewha Womans University School of Medicine and Ewha Medical Research Institute, Seoul, Republic of Korea

^*^Co-correspondence

**Contents**

**Table S1.** Detailed description on district-level indicators

**Table S2.** Descriptive statistics of exposures and regional indicators in 229 districts

**Table S3.** Number of preterm births by regional characteristics

**Table S4.** Number of preterm births by individual and regional characteristics in rural and urban

**Table S5.** Sensitivity analysis with different time window of exposure to ambient temperature

**Table S6.** Sensitivity analysis without PM_10_ adjustment in a conditional logistic model

**Fig. S1.** Time-stratified case-crossover design

**Fig. S2.** Time trend of proportion of preterm birth (PTB) during 2011–2019

**Fig. S3.** Spatial distribution of district-level mean temperature and PM_10_ concentration during 2011–2019

**Table S1.** Detailed description on district-level indicators

|  | Definition | Collection period | Data source |
| --- | --- | --- | --- |
| Population density (/km^2^) | Number of population / Area (km^2^) | 2011–2019 | Database of community health outcomes and determinants distributed by the Korea Diseases Control and Prevention Agency (KCDA) |
| Park area per person (m^2^) | All parks prescribed by ordinance were considered. | 2011–2019 | Database of community health outcomes and determinants distributed by the KCDA |
| Enhanced vegetation index (EVI) | EVI was obtained with a 1km × 1km spatial resolution and averaged across each district for the analysis. | 2011–2019 | Moderate Resolution Imaging Spectroradiometer (MODIS) (MOD13A2 v006) |
| # OB/GYN specialists per 1,000 persons | Number of obstetrics and gynecology (OB/GYN) specialists per 1,000 persons. | 2011–2019 | Database of community health outcomes and determinants distributed by the KCDA |
| ER utilization rate within standard time (%) | Percentage of utilization within standard time (60 minutes) out of total emergency room utilization by local residents. | 2011–2019 | Database of community health outcomes and determinants distributed by the KCDA |
| DR utilization rate within standard time (%) | Percentage of utilization within standard time (60 minutes) out of total delivery room utilization by local residents. | 2011–2019 | Database of community health outcomes and determinants distributed by the KCDA |

DR=delivery room, ER=emergency room, OB/GYN=obstetrics and gynecology

**Table S2.** Descriptive statistics of exposures and regional indicators in 229 districts during 2011­–2019

|  | Mean±SD | Min. | Q1 | Q2 | Q3 | Max. |
| --- | --- | --- | --- | --- | --- | --- |
| **Exposure variable** |  |  |  |  |  |  |
| Temperature (℃) | 12.81±1.29 | 8.84 | 12.13 | 12.91 | 13.60 | 16.45 |
| PM_10_ concentration (µg/m^3^) | 42.08±7.26 | 19.82 | 38.02 | 42.61 | 46.64 | 62.16 |
| **District-level indicator** |  |  |  |  |  |  |
| Population density (/km^2^) | 3958.08±6232.03 | 19.77 | 97.41 | 476.89 | 5827.13 | 27647.03 |
| Park area per person (m^2^) | 21.81±20.71 | 0.00 | 8.61 | 17.87 | 26.53 | 136.45 |
| Enhanced vegetation index (EVI) | 2831±687 | 1018 | 2326 | 3062 | 3359 | 3771 |
| # OB/GYN specialists per 1,000 persons |  |  |  |  |  |  |
| ER utilization rate  within standard time (%) | 47.31±30.32 | 0.00 | 18.54 | 48.47 | 77.98 | 91.16 |
| DR utilization rate  within standard time (%) | 64.13±34.68 | 0.00 | 34.14 | 82.79 | 93.08 | 96.58 |

DR=delivery room, ER=emergency room, OB/GYN=obstetrics and gynecology

**Table S3.** Number of preterm births by regional characteristics (PTB, n=160,067)

|  | N | % |
| --- | --- | --- |
| Urbanization |  |  |
| Rural | 27,689 | 17.30 |
| Urban | 132,378 | 82.70 |
| Park area per person |  |  |
| Low | 69,183 | 43.22 |
| Mid | 53,959 | 33.71 |
| High | 36,925 | 23.07 |
| Enhanced vegetation index (EVI) |  |  |
| Low | 80,892 | 50.54 |
| Mid | 57,007 | 35.61 |
| High | 20,181 | 12.61 |
| # OB/GYN specialists per 1,000 persons |  |  |
| Low | 34,112 | 21.31 |
| Mid | 67,411 | 42.11 |
| High | 58,544 | 36.57 |
| ER utilization rate within standard time |  |  |
| Low | 11,398 | 7.12 |
| Mid | 61,274 | 38.28 |
| High | 87,395 | 54.60 |
| DR utilization rate within standard time |  |  |
| Low | 9,700 | 6.06 |
| Mid | 66,347 | 41.45 |
| High | 84,020 | 52.49 |

Note: Urban and rural were categorized using median of population density.
DR=delivery room, ER=emergency room, OB/GYN=obstetrics and gynecology

**Table S4.** Number of preterm births by individual and regional characteristics in rural and urban (PTB, n=160,067)

|  | N (%) | |
| --- | --- | --- |
|  | Rural  (n=27,689) | Urban  (n=132,378) |
| **Individual characteristics** | | |
| **Maternal age** |  |  |
| < 35 years | 19,848 (71.68) | 92,476 (69.86) |
| ≥ 35 years | 7,841 (28.31) | 39,902 (30.14) |
| **Parity** |  |  |
| Primiparity | 12,501 (45.15) | 67,818 (51.23) |
| Multiparity | 10,426 (37.65) | 49,857 (37.66) |
| **Maternal education level** |  |  |
| ≤ College | 26,370 (95.24) | 121,736 (91.96) |
| > College | 1,196 (4.32) | 9,774 (7.38) |
| **Classification of prematurity^a^** |  |  |
| Late to moderate preterm | 24,733 (89.32) | 117,937 (89.09) |
| Very to extreme preterm | 2,956 (10.68) | 14,441 (10.91) |
| **Regional characteristics** | | |
| **Park area per person** |  |  |
| Low | 13,790 (49.80) | 65,074 (49.16) |
| High | 13,899 (50.20) | 67,304 (50.84) |
| **Enhanced vegetation index (EVI)** |  |  |
| Low | 12,822 (46.31) | 64,948 (49.06) |
| High | 12,880 (46.52) | 67,430 (50.94) |
| **# OB/GYN specialists per 1,000 persons** |  |  |
| Low | 13,470 (48.65) | 65,890 (49.77) |
| High | 14,219 (51.35) | 66,488 (50.23) |
| **ER utilization rate within standard time** |  |  |
| Low | 13,745 (49.64) | 66,126 (49.95) |
| High | 13,944 (50.36) | 66,252 (50.05) |
| **DR utilization rate within standard time** |  |  |
| Low | 13,704 (49.49) | 65,947 (49.82) |
| High | 13,985 (50.51) | 66,431 (50.18) |

Note: Urban and rural were categorized using median of population density.
DR=delivery room, ER=emergency room, OB/GYN=obstetrics and gynecology

**Table S5.** Sensitivity analysis with different time window of exposure to ambient temperature: association between ambient temperature during the last three and five weeks before delivery and preterm birth by individual characteristics

|  | OR (95% CI) | | |
| --- | --- | --- | --- |
|  | 3 weeks | 5 weeks |  |
| **Overall** | 1.02 (1.01, 1.04) | 1.04 (1.02, 1.06) |  |
| **Maternal age** |  |  |  |
| < 35 years | 1.01 (0.99, 1.03) | 1.03 (1.01, 1.05) |  |
| ≥ 35 years | 1.07 (1.03, 1.11) | 1.09 (1.06, 1.13) |  |
| **Parity** |  |  |  |
| Primiparity | 1.02 (1.00, 1.05) | 1.04 (1.02, 1.07) |  |
| Multiparity | 1.04 (1.02, 1.07) | 1.05 (1.03, 1.08) |  |
| **Maternal education level** |  |  |  |
| ≤ College | 1.03 (1.01, 1.04) | 1.04 (1.03, 1.06) |  |
| > College | 1.01 (0.95, 1.08) | 1.01 (0.95, 1.07) |  |
| **Classification of prematurity^a^** |  |  |  |
| Late to moderate preterm | 1.02 (1.01, 1.04) | 1.04 (1.02, 1.06) |  |
| Very to extreme preterm | 1.03 (0.98, 1.09) | 1.06 (1.00, 1.12) |  |

Note: ORs were presented as those for 5℃ increase in moving averaged-temperature during the last three or five weeks before delivery.
^a^Classification of prematuriy was specified based on gestational age: late to moderate preterm (32 to 37 weeks) and very to extreme preterm (20 to 32 weeks).
CI=confidence interval, OR=odds ratio

**Table S6.** Sensitivity analysis without PM_10_ adjustment in a conditional logistic model: association between ambient temperature during the last four weeks before delivery and preterm birth by individual characteristics

|  | OR (95% CI) | |
| --- | --- | --- |
| **Overall** | 1.04 (1.02, 1.05) |  |
| **Maternal age** |  |  |
| < 35 years | 1.03 (1.01, 1.05) |  |
| ≥ 35 years | 1.06 (1.03, 1.09) |  |
| **Parity** |  |  |
| Primiparity | 1.04 (1.02, 1.06) |  |
| Multiparity | 1.03 (1.01, 1.06) |  |
| **Maternal education level** |  |  |
| ≤ College | 1.05 (1.03, 1.06) |  |
| > College | 1.02 (0.96, 1.09) |  |
| **Classification of prematurity^a^** |  |  |
| Late to moderate preterm | 1.04 (1.02, 1.05) |  |
| Very to extreme preterm | 1.04 (0.99, 1.09) |  |

Note: ORs were presented as those for 5℃ increase in moving averaged-temperature during the last four weeks before delivery.
^a^Classification of prematuriy was specified based on gestational age: late to moderate preterm (32 to 37 weeks) and very to extreme preterm (20 to 32 weeks).
CI=confidence interval, OR=odds ratio

**Fig. S1.** Time-stratified case-crossover design


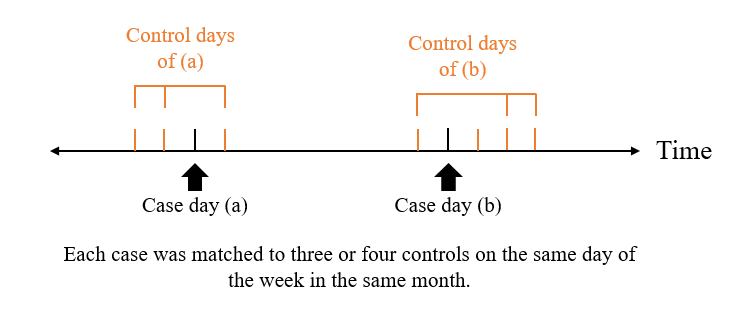


**Fig. S2.** Time trend of proportion of preterm birth (PTB) and temperature during 2011–2019. Proportion of PTB was calculated by dividing the number of PTB by total number of births.


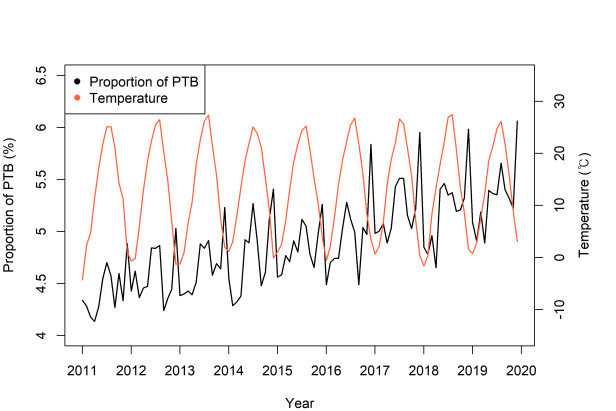


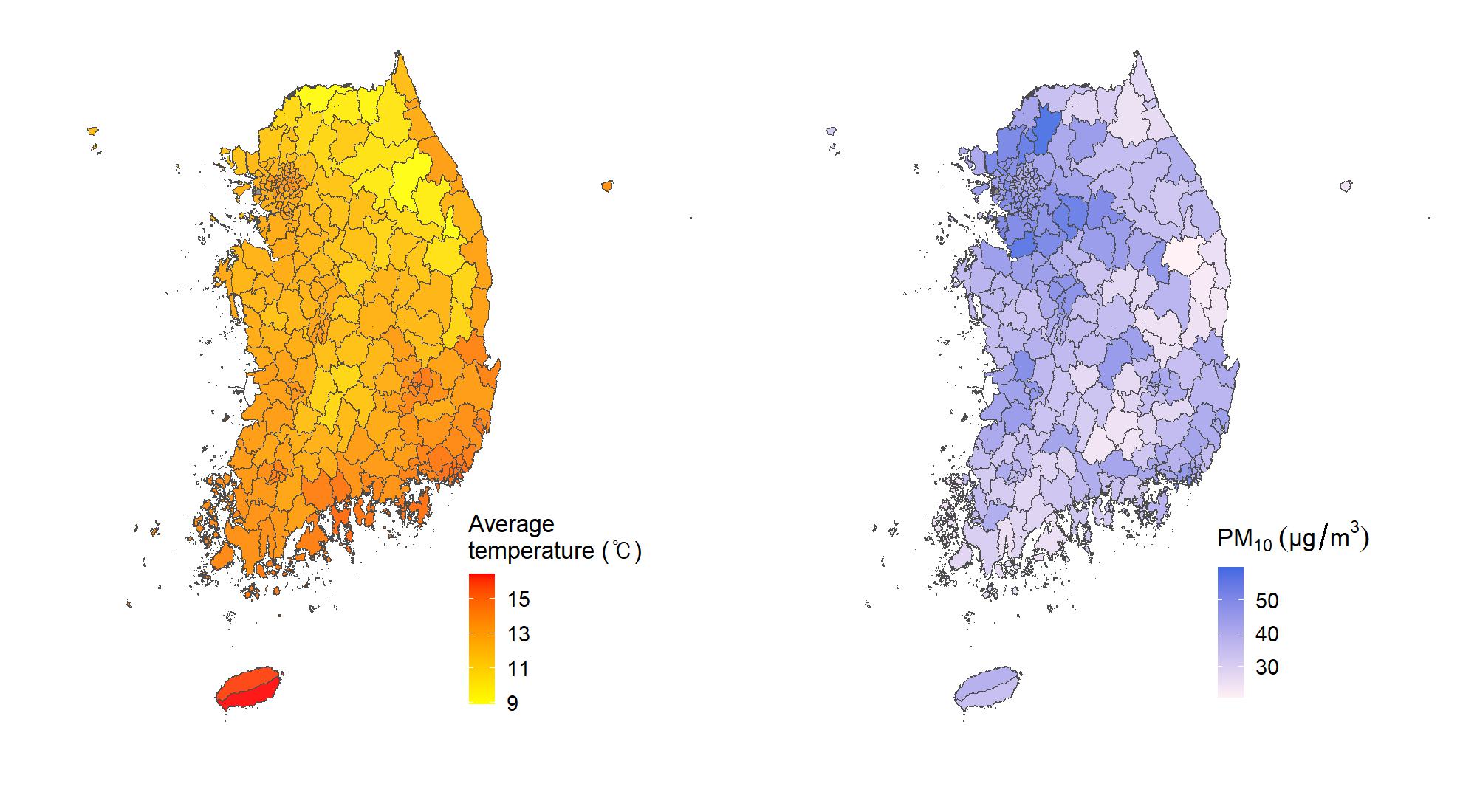
**Fig. S3.** Spatial distribution of district-level mean temperature and PM_10_ concentration during 2011–2019
